# Intergenerational Dental Fear and Anxiety: Children's Pattern of Dental Service Use

**DOI:** 10.1111/cdoe.70057

**Published:** 2026-02-11

**Authors:** Helena Silveira Schuch, Cinthia Fonseca Araujo, Giulia Tarquinio Demarco, Mariana Gonzalez Cademartori, Marília Leão Goettems, Andréa Dâmaso Bertoldi, Daniel W. McNeil, Flávio Fernando Demarco

**Affiliations:** ^1^ School of Dentistry, Faculty of Health, Medical and Behavioural Sciences The University of Queensland Brisbane Australia; ^2^ Postgraduate Program in Dentistry Federal University of Pelotas Pelotas Brazil; ^3^ Postgraduate Program in Epidemiology Federal University of Pelotas Pelotas Brazil; ^4^ College of Dentistry University of Florida Gainesville Florida USA

**Keywords:** dental anxiety, dental care, healthcare disparities, longitudinal studies, mother–child relations, paediatric dentistry

## Abstract

**Objectives:**

Dental care‐related fear and anxiety, involving emotional reactions to dental situations, can negatively impact children's quality of life, oral and systemic health. Recognising maternal characteristics as key determinants of child health, this study examined the intergenerational relationship between dental fear/anxiety and early childhood dental service utilisation.

**Methods:**

Data were drawn from the baseline (birth), 12‐month, and four‐year follow‐ups of the 2015 Pelotas Birth Cohort Study (Brazil). Outcomes included child dental fear/anxiety and dental service use (frequency and reasons) at age four. Exposures were maternal dental fear/anxiety at 12‐month and maternal assessment of child dental fear/anxiety. Covariates, including maternal education and family income, were collected perinatally. All variables were obtained through questionnaires administered to mothers at each follow‐up. Analyses used Poisson and multinomial logistic regression models.

**Results:**

The sample included 3809 mother–child dyads; 79.7% of mothers and 64.2% of children had no reported dental fear/anxiety, yet 64.0% of children had never visited a dentist. Adjusted analyses showed that maternal dental fear/anxiety was associated with a 1.20 times higher prevalence of childhood dental fear/anxiety (PR = 1.20, 95% CI = 1.09; 1.32). Children with dental fear/anxiety had a 1.89 times higher prevalence of visits for curative reasons (PR = 1.89, 95% CI = 1.45; 2.48) and a 2.10 times higher prevalence of never visiting a dentist (PR = 2.10, 95% CI = 1.78; 2.48), adjusting for maternal dental fear/anxiety.

**Conclusion:**

Early and regular preventive visits may help mitigate dental fear/anxiety. This study highlights the intergenerational link between maternal and childhood dental fear and its impact on dental service utilisation.

## 1 Introduction

The first thousand days in a child's development, which comprise the woman's pregnancy and her child's first two years of life, are recognised as a crucial interval for health and development. Actions and influences in this period have a high impact on a child's growth and well‐being [1]. In this context, early prevention is crucial for short‐ and long‐term outcomes.

Dental care‐related fear and anxiety are defined as negative feelings associated with the dental setting [2, 3]. Referred to here as ‘dental fear’ for the sake of simplicity, this condition can negatively impact oral health due to the person's lack of cooperation towards oral health care. A vicious cycle has long been proposed ([4, 5, 6]), where individuals avoid or delay dental visits because of dental fear. This leads to the development or exacerbation of dental issues, requiring more invasive and traumatic procedures, which may, in turn, increase dental fear and develop an avoidant behaviour concerning oral health care [7, 8]. Clinically significant levels of dental fear and anxiety can be manifested across the lifespan, but prevention and treatment efforts in childhood are particularly important because of the potential impacts lifelong [8, 9]. Due to the dental fear experience itself and all its potential ramifications, dental fear can have a detrimental effect on children's dental and general health [10]. When childhood dental fear is identified and managed properly, the progression of this condition into adulthood can be prevented or minimised [11, 12].

Maternal characteristics, as mothers are usually the primary caregivers, are determinants in a child's health and shape a child's experience. A recently published birth cohort study with Finnish families observed the highest number of preventive procedures among children from mothers reporting stable low dental anxiety, but this pattern was not identified when they assessed the father's trajectory of dental anxiety [13]. Nevertheless, fathers also have an important role in a child's dental fear [14].

Maternal mental health, including conditions such as depression, can have a negative effect on children since mothers are generally the main models for learning health‐related behaviours [7]. In addition to the emotional issues faced by caregivers, mothers' perception of their children's oral health and the behavioural patterns of care for their oral health may also influence their decision to take their children to dental appointments [15, 16]. A recent study in this same cohort sought to evaluate factors associated with the first dental visit in the first year of life of children and demonstrated that mothers who visited the dentist for preventive reasons were more likely to take their children to visits, while those with some tooth loss due to caries were not [16]. As aforementioned, the dental fear of caregivers can impact children's oral health, as it can have repercussions for their own oral health as well [13, 17]. The socioeconomic context in which a child is inserted also plays a decisive role, as those in vulnerable backgrounds tend to face barriers that delay access to dental care, increasing the chances of early negative experiences [18, 19].

The exploration of dental service use aligns with Andersen's Behavioural Model, which conceptualises health service utilisation as a key outcome influenced by predisposing, enabling, and need factors, ultimately contributing to health outcomes [20]. Within this framework, understanding patterns of dental service utilisation is essential to capture how behavioural and contextual factors, such as dental fear, shape oral health trajectories. In the context of the vicious dental fear cycle ([4, 5, 6]), service utilisation represents a critical link between psychological determinants and oral health outcomes, where avoidance driven by fear can perpetuate disease progression and reinforce fear over time. Evaluating this relationship from an intergenerational perspective is particularly important, as parental dental fear may influence children's attitudes and behaviours towards oral health care. Addressing this gap, the present study aimed to examine the relationship between intergenerational dental fear and dental service use among participants in the 2015 Pelotas Birth Cohort study.

## 2 Methodology

This manuscript is structured following the STROBE guidelines [21].

Data were drawn from the 2015 Pelotas Birth Cohort Study (hereafter referred to as the 2015 Pelotas Study). Pelotas is a middle‐sized city in the southernmost state of Brazil. Every 11 years, a new prospective study starts in the town, being eligible to the study all children born in the city in that year and whose mothers live in the urban area of Pelotas or the city's fishing colony. The first of the Pelotas Birth Cohort Studies started in 1982, and all studies are ongoing. To allow comparability with the previous editions of the study, the 2015 Pelotas Study also included children from a suburb of a neighbouring town, which previously was within the administrative area of Pelotas.

The study protocol was approved by the Human Research Ethics Committees from the Federal University of Pelotas (approval number: CAAE registration number: 26746414.5.0000.5313). A signed informed consent form was obtained from all mothers' participants and from the child's primary caregiver at baseline and follow‐ups.

From the first of January to the 31st of December of 2015, a research team visited all hospitals in the city daily to identify and recruit eligible participants. Of the 5598 children born in Pelotas in that year, 4387 were eligible for the study and comprised the target population. After excluding stillborn babies (*n* = 54), refusals (*n* = 51), and deliveries not captured by the study teams (*n* = seven), 4275 children were the baseline of the 2015 Birth Cohort, representing 98.7% of the target population. Until now, there have been eight follow‐up assessments with this cohort: (a) a perinatal study with the newborns, (b) at 3 months, (c) at 12 months, (d) at 2 years, (e) at 4 years, (f) at 5 years, (g) at 6–7 years, and (h) at 9 years. A subsample of the mothers was also included in a prenatal study. The methodological aspects of this cohort study have been previously described [22, 23].

Data collection included interviews with the primary caregiver and physical and cognitive evaluations of the children. A trained and calibrated research team performed data collection at each follow‐up, using validated instruments and standardised questionnaires. Furthermore, a child clinical oral health examination was carried out at the follow‐up at 4 years of age by 12 previously trained and calibrated dentists. A pilot study was carried out in each follow‐up and quality control was performed by re‐interviewing 10% of the sample and assessing consistency between their answers. In the present study, we used data from the baseline (perinatal), and the follow‐ups at 12 months, and 4 years.

This study has two outcomes: child dental fear and pattern of use of dental services, both collected at 4 years. Child dental fear was measured by the following question, asked to the mother or primary caregiver: ‘Do you think that (child's name) is or would be afraid of going to the dentist?’ This variable was dichotomized in the presence of fear (yes, a little; yes; and yes, a lot) and absence of fear (no).

The pattern of dental services was reflected by the frequency of the use of the services and the reason for these visits measured by the questions: ‘Has (child's name) ever visited a dentist, apart from the dentist from this study?’ (yes/no) and ‘What was the reason for the last dental visit’ with the options ‘routine or preventive visit’, ‘dental pain’, or ‘treatment’. The pattern of dental services was reflected by the frequency of the use of the services and the reason for these visits measured by the questions: ‘Has (child's name) ever visited a dentist, apart from the dentist from this study?’ (yes/no) and ‘What was the reason for the last dental visit’ with the options ‘routine or preventive visit’, ‘dental pain’, or ‘treatment’. The combined option ‘routine or preventive visit’ was intended to capture non–symptom‐driven, health‐oriented attendance, such as regular check‐ups or preventive care, distinguishing it from visits motivated by dental problems. For analytical purposes, the reason for the last dental visit was dichotomized as preventive versus curative (treatment or dental pain), and both variables, frequency and reason, were combined. Through the combination of these dichotomous variables, a variable of use of services was created, with categories ‘the child has been to the dentist, and the last appointment was for preventive reasons”, ‘the child has been to the dentist, and the last appointment was curative’, and ‘the child has never been to the dentist’.

These two outcomes were analysed separately. The first, child dental fear, represents an emotional or psychological construct, while the second, pattern of dental service utilisation, reflects a behavioural outcome capturing both access and purpose of dental care.

The study's exposures were maternal dental fear and maternal perception of child dental fear. Maternal dental fear was assessed at the child's 12‐month follow‐up through the Dental Anxiety Question (DAQ): ‘Are you afraid of going to the dentist?’ [24], with potential answers ‘No’, ‘Yes, a little’, ‘Yes’, and ‘Yes, a lot’. For analytical purposes, this information was dichotomized as no versus yes (yes, a little/yes/yes, a lot). Maternal perception of child dental fear was both an exposure and an outcome. It was an exposure to the association of child dental fear and child use of dental services, and an outcome for the exposure maternal dental fear. Child dental fear was measured at 4 years, as previously described.

Covariates were collected at the perinatal wave and included maternal education, collected as years of formal education, and family income, collected as the sum of family earnings in the previous month and categorised according to the Brazilian government's monthly minimum wage (MMW). The amount of monthly family income was calculated as a value relative to MMW in five categories as follows: (a) less than the MMW, (b) one to three times the MMW, (c) more than three to six times the MMW, (d) more than six to ten times the MMW, and (e) more than ten times the MMW.

Statistical analyses included the evaluation of absolute and relative frequencies of the variables of interest. For the analyses of the association between maternal and child dental fear, Poisson regression models were used. For the analyses of the association between child dental fear and pattern of dental services use, multinomial logistic regression models were used, due to the nature of the outcome. Data analyses were performed in the software Stata.

## 3 Results

A total of 3809 mother–child dyads with complete data were included in the analytical sample, which was comparable to the full sample (Table 1). Most mothers had more than 9 years of education (65.8%) and about half reported a family income between one and three times the minimum monthly wage. Dental fear was reported by 20.3% of mothers, and mothers indicated that 35.8% of their children exhibited dental fear. Two‐thirds of the children had never been to a dental visit, and among those who had, most attended for preventive rather than curative reasons.

**TABLE 1:** Characteristics and comparison between the full and the analytical samples.

	Full sample *N* = 4275		Analytical sample *N* = 3809	
	%	95% CI	%	95% CI
*Maternal education*				
12 years or more	31.1	29.7; 32.5	30.6	29.1; 32.1
9 to 11 years	34.1	32.7; 35.5	35.2	33.7; 36.8
5 to 8 years	25.6	24.3; 27.0	25.6	24.2; 27.0
0 to 4 years	9.2	8.3; 10.0	8.6	7.7; 9.5
*Family income (monthly minimum wages)*				
> 10	6.3	5.6; 7.1	6.0	5.3; 6.8
6.1–10	7.6	6.8; 8.4	7.3	6.5; 8.1
3.1–6	26.4	25.1; 27.7	26.7	25.3; 28.1
1–3	47.1	45.6; 48.6	47.8	46.2; 49.4
≤ 1	12.6	11.6; 13.6	12.2	11.2; 13.3
*Maternal dental fear*				
No	79.6	78.4; 80.8	79.7	78.4; 81.0
Yes, a little	7.1	6.3; 7.9	7.0	6.3; 7.9
Yes	3.5	2.9; 4.1	3.5	2.9; 4.1
Yes, a lot	9.8	9.0; 10.8	9.8	8.9; 10.7
*Child dental fear*				
No	63.0	61.5; 64.5	64.2	62.7; 65.7
Yes, a little	22.2	21.0; 23.5	22.2	20.9; 23.6
Yes, a lot	13.5	12.4; 14.6	13.6	12.5; 14.7
Doesn’t know	1.3	1.0; 1.7	—	—
*Has the child ever been to a dental visit?*				
Yes	35.6	34.1; 37.1	36.0	34.5; 37.6
No	64.4	62.9; 65.9	64.0	62.4; 65.5
*Reason for last dental visit*				
Preventive	76.7	74.4; 78.8	76.4	74.1; 78.6
Curative (treatment or dental pain)	23.3	21.2; 25.6	23.6	21.4; 25.9
*Combined dental visit pattern*				
Have been, last visit was preventive	27.3	25.9; 28.7	27.5	26.1; 29.0
Have been, last visit curative	8.3	7.5; 9.2	8.5	7.7; 9.4
Has never been	64.4	63.0; 65.9	64.0	62.4; 65.5

*Note:* 2015 Pelotas birth cohort study.

As shown in Table 2, both lower maternal education and family income were associated with a higher proportion of children who had never visited the dentist. Children of mothers with more education or higher income were more likely to have preventive visits. Dental fear also showed a clear pattern: mothers and children without dental fear were more likely to attend preventive visits, whereas higher fear levels corresponded to fewer or more problem‐driven visits.

**TABLE 2:** Pattern of child dental visit by sociodemographic and dental fear variables.

	Visited for prevention		Visited for curative		Never visited		*p*
	*n*	Row%	*n*	Row%	*n*	Row%	
*Maternal education*							< 0.001
12 years or more (completed high school)	480	41.2 (38.4; 44.1)	109	9.4 (7.8; 11.2)	576	49.4 (46.6; 52.3)	
9 to 11 years	340	25.3 (23.1; 27.7)	117	8.7 (7.3; 10.3)	885	65.9 (63.4; 68.4)	
5–8 years	168	17.2 (15.0; 19.7)	77	7.9 (6.4; 9.8)	730	74.9 (72.0; 77.5)	
0–4 years	61	18.7 (14.8; 23.2)	21	6.4 (4.2; 9.6)	245	74.9 (69.9; 79.3)	
*Family income (minimum wages)*							< 0.001
> 10	135	58.7 (52.2; 64.9)	20	8.7 (5.7; 13.1)	75	32.6 (26.9; 38.9)	
6.1–10	117	42.2 (36.5; 48.1)	28	10.1 (7.1; 14.2)	132	47.7 (41.8; 53.5)	
3.1–6	306	30.1 (27.3; 33.0)	92	9.0 (7.4; 11.0)	619	60.9 (57.8; 63.8)	
1–3	391	21.5 (19.7; 23.4)	156	8.6 (7.4; 9.9)	1273	69.9 (67.8; 72.0)	
≤ 1	100	21.5 (18.0; 25.5)	28	6.0 (4.2; 8.6)	337	72.5 (68.2; 76.3)	
*Maternal dental fear*							0.002
No	878	28.9 (27.3; 30.6)	255	8.4 (7.5; 9.4)	1903	62.7 (60.9; 64.4)	
Yes, a little	65	24.3 (19.5; 29.7)	17	6.3 (4.0; 10.0)	186	69.4 (63.6; 74.6)	
Yes	33	24.8 (18.2; 32.8)	10	7.5 (4.1; 13.4)	90	67.7 (59.3; 75.1)	
Yes, a lot	73	19.6 (15.9; 24.0)	42	11.3 (8.4; 14.9)	257	69.1 (64.2; 73.6)	
*Child dental fear*							< 0.001
No	804	32.9 (31.0; 34.8)	202	8.3 (7.2; 9.4)	1440	58.9 (56.9; 60.8)	
Yes, a little	171	20.2 (17.6; 23.0)	68	8.0 (6.3; 10.1)	608	71.8 (68.6; 74.7)	
Yes, a lot	74	14.3 (11.6; 17.6)	54	10.5 (8.1; 13.4)	388	75.2 (71.3; 78.7)	

*Note:* 2015 Pelotas birth cohort study. *N* = 3809.

In the adjusted Poisson regression models (Table 3), maternal dental fear was positively associated with child dental fear, even after controlling for maternal education (PR = 1.20; 95% CI = 1.09; 1.32).

**TABLE 3:** Associations between maternal and child dental fear.

Maternal dental fear	Presence of child dental fear^a^			
	Crude		Adjusted^b^	
	PR	95% CI	PR	95% CI
No (reference)	1.0		1.0	
Yes+	1.25	1.14; 1.38	1.20	1.09; 1.32

*Note:* Poisson regression analysis. 2015 Pelotas birth cohort study. *N* = 3809.

^a^ Reference absence of child dental fear.

^b^ Adjusted for maternal education in number of years.

As shown in Table 4, children with dental fear were substantially more likely to have never visited the dentist or to have visited for curative rather than preventive reasons. After adjusting for maternal dental fear, maternal education, and family income, children with dental fear remained about twice as likely to have never visited (PR = 2.10; 95% CI = 1.78; 2.48) or to have attended for curative purposes (PR = 1.89; 95% CI = 1.45; 2.48).

**TABLE 4:** Association between child dental fear and pattern of dental visit.

Child dental fear	Child pattern of dental visit^a^							
	Crude				Adjusted^b^			
	Visited for curative reasons		Never visited		Visited for curative reasons		Never visited	
	PR	95% CI	PR	95% CI	PR	95% CI	PR	95% CI
No (reference)	1.0		1.0		1.0		1.0	
Yes+	1.98	1.52; 2.59	2.27	1.93; 2.67	1.89	1.45; 2.48	2.10	1.78; 2.48

*Note:* Multinomial logistic regression. 2015 Pelotas birth cohort study. *N* = 3809.

^a^ Reference visited for preventive reasons.

^b^ Adjusted for maternal dental fear, maternal education in number of years, and family income in minimum wages.

## 4 Discussion

This cohort study, comprising a large sample of mothers and children prospectively evaluated, demonstrated that the presence of dental fear in mothers significantly increases the prevalence of dental fear in children. Furthermore, the study found that dental fear in children is associated with a higher prevalence of dental visits for curative reasons or a complete absence of dental visits. The findings support an intergenerational effect on dental fear and underscore the importance of a comprehensive approach to effectively promote oral health.

The findings are supported by and corroborate previous literature. A recent systematic review and meta‐analysis reported a prevalence of significant levels of dental fear similar to those observed in the present study, approximately 30% in children aged 2–6 years, with higher risks observed in children who do not have regular dental visits and those with a history of tooth decay [25]. In addition, a structured review and meta‐analysis already confirmed the relationship between parental and child dental fear [10]. Studies conducted with Finnish families further demonstrated a strong association between dental fear in children and that of other family members [26], as well as maternal dental fear and patterns of child dental visits [13]. Among the findings of a cross‐sectional study, dental fear was associated with dental visits for symptomatic reasons, previous experience of pain, and the tendency to avoid dental visits due to this feeling [27]. Additionally, the literature suggests that dental fear is associated with missed dental visits and with the presence of dental caries and reports maternal dental fear as an important etiological factor for childhood dental fear [3, 14, 28].

Regular dental visits for preventive care in children have been proven to significantly reduce the chances of invasive treatments and negative experiences later on [29]. In addition, it has been observed that children with dental fear or previous negative experiences regarding dental visits require greater support and attention [30]. Therefore, efforts for early identification and management of dental fear are crucial for interrupting the dental fear cycle and promoting healthier outcomes. This is particularly crucial given the association between dental fear and poor oral health, which demonstrates the importance of professionals and families acting together considering these questions related to children [12]. A study with data from the 2004 Pelotas Birth Cohort Study demonstrated that the presence of carious lesions and dental pain was associated with dental fear, regardless of socioeconomic status or dental service utilisation [31]. Monitoring dental fear during childhood is strongly recommended, aiming at the implementation of measures to prevent its progression into adulthood, being a valuable objective for public health interventions [11, 31].

Maternal characteristics exert a significant influence on child health outcomes. A study carried out in the same city as the present sample showed that children whose mothers have a low level of education and who do not regularly visit the dentist have an increased risk of receiving inadequate dental care themselves [15]. Therefore, the importance of targeting factors related to the mothers, such as education and income, extends beyond their own life experiences and health but can also benefit the health and healthcare pattern of their children [17].

This study has some limitations that should be considered when interpreting the results. First, all data were collected solely through maternal or caregiver reports, both those referring to themselves and those referring to their children. This is particularly relevant for the assessment of child dental fear, which was measured using a question that may reflect either an actual fear based on previous dental experience or a parental expectation of how the child might react in a dental context. In addition, at the developmental stage of 4 years, children are still learning to articulate their emotions [32], making it difficult to determine the extent to which maternal reports may have led to underreporting or overreporting of children's dental fear. Previous research has shown that parents do not always reliably assess their children's dental fear [33, 34], this necessitates cautious interpretation of our findings due to the potential for misclassification bias. Also, fathers were not included in this study; they have an important influence on dental fear [3, 14, 35]. Secondly, it was not possible to definitively ascertain whether the non‐occurrence of dental visits was from challenges in accessing dental services or from other reasons. Even though Brazil has free‐of‐charge dental care as part of the public health system, including preventive and curative services for children, the proportion of children in this age group who have never visited a dentist remains high, reflecting potential barriers. It is important to consider that patterns of dental service utilization at this age primarily reflect parental behavior, given the limited autonomy that children have over health‐related decisions. In addition, although dental care‐related anxiety and fear are dimensional and not binary conditions [3], as it is a large‐scale epidemiological study, categorical analyses were used to explicate these data. This dichotomization may have resulted in some loss of information. Moreover, these data were collected using a single‐item question, which, while a simple and valid measure of overall dental anxiety [24], may not fully capture the complexity of this question. Finally, the inclusion of other variables as confounding factors in the models could have influenced the estimates. Although efforts were made to control for relevant factors, the possibility of unmeasured variables influencing the results cannot be entirely ruled out.

All that said, this study has several strengths that provide robustness to its findings. Among these strengths is the quality of the sample, which comprises a large sample of individuals born in the city in 2015. Through comprehensive data collection from pregnancy to periodic follow‐ups, this study offers a representative outlook of the population's health dynamics. In addition, it is a cohort study, a study design that is considered the gold standard in epidemiological research [36]. The combination of a large, representative sample and a rigorous methodology lends considerable credence to the findings. This study included children at age four and provided evidence of the intergenerational impact of dental fear at an early developmental age. It extends existing literature that has primarily focused on older children and adolescents [14, 35], highlighting that maternal dental fear can already influence children's emotional responses to dental care before regular service use is established.

These findings have important implications for both clinical practice and public health policy. From a clinical perspective, they underscore the importance of adopting a family‐centred approach in dental care, where parental attitudes and fears are acknowledged as integral components of the child's oral health experience. Early identification of maternal or caregiver dental fear may allow dental professionals to provide tailored support, positive conditioning, and anticipatory guidance to reduce the risk of transmitting fear to children.

From a public health perspective, the results point to the need to strengthen preventive and family‐oriented oral health programs, particularly during early childhood. Strategies such as incorporating caregiver education into early dental visits, community health promotion, and antenatal or early parenting programs could help prevent the establishment of dental fear and promote positive dental care trajectories.

Moreover, it is important to consider factors such as the socioeconomic status of the families and maternal education. As evidenced in this study, these factors can influence the patterns and absence of dental visits and, for that reason, interventions aimed at improving access to dental care should address underlying socioeconomic disparities to ensure equitable oral health outcomes.

## 5 Conclusion

This cohort study suggests that the presence of high levels of maternal dental fear is associated with a greater prevalence of childhood dental fear. Additionally, the presence of significant dental fear in children is associated with a higher prevalence of never having visited a dentist or having visited for curative reasons. These results should be interpreted with caution, given the use of maternal proxy reports and other methodological limitations. However, it highlights the importance of considering dental care‐related fear and anxiety in children and their caregivers, and implementing comprehensive oral health measures that involve the entire family. Efforts should be directed towards promoting and raising awareness of the importance of preventive dental visits.

## Funding

This article is based on data from the study ‘Pelotas Birth Cohort, 2015’ conducted by Postgraduate Program in Epidemiology at Universidade Federal de Pelotas, with the collaboration of the Brazilian Public Health Association (ABRASCO). The first phases of the 2015 Pelotas (Brazil) Birth Cohort was funded by the Wellcome Trust (095582, 10735_Z_18_Z). Funding for specific follow‐up visits was also received from the Conselho Nacional de Desenvolvimento Científico e Tecnológico (CNPq) (426230/2018‐3, 54796/2014‐5) and Fundação de Amparo a Pesquisa do Estado do Rio Grande do Sul (FAPERGS) and Children’s Pastorate sponsored follow‐up at twenty‐four months; and FAPERGS—PPSUS and the Bernard van Leer Foundation (BRA‐2018‐178) for the 4‐year follow‐up. At the 4 years follow‐up the 2015 cohort also was funded by the Department of Science and Technology (DECIT/Brazilian Ministry of Health). Daniel W. McNeil’s involvement in this article was supported in part by National Institute of Dental and Craniofacial Research U01 DE033978, U01 DE033262, and UG3/UH3 DE032004. CNPQ PRONEX: 12/2014 Grantnumber16.0471‐4.

## Conflicts of Interest

The authors declare no conflicts of interest.

## Data Availability

The data that support the findings of this study are available on request from the corresponding author. The data are not publicly available due to privacy or ethical restrictions.
